# DNA End Resection Controls the Balance between Homologous and Illegitimate Recombination in *Escherichia coli*

**DOI:** 10.1371/journal.pone.0039030

**Published:** 2012-06-14

**Authors:** Siniša Ivanković, Damir Đermić

**Affiliations:** 1 Division of Molecular Medicine, Ruđer Bošković Institute, Zagreb, Croatia; 2 Division of Molecular Biology, Ruđer Bošković Institute, Zagreb, Croatia; University of Massachusetts, United States of America

## Abstract

Even a partial loss of function of human RecQ helicase analogs causes adverse effects such as a cancer-prone Werner, Bloom or Rothmund-Thompson syndrome, whereas a complete RecQ deficiency in *Escherichia coli* is not deleterious for a cell. We show that this puzzling difference is due to different mechanisms of DNA double strand break (DSB) resection in *E. coli* and humans. Coupled helicase and RecA loading activities of RecBCD enzyme, which is found exclusively in bacteria, are shown to be responsible for channeling recombinogenic 3′ ending tails toward productive, homologous and away from nonproductive, aberrant recombination events. On the other hand, in *recB1080/recB1067* mutants, lacking RecBCD’s RecA loading activity while preserving its helicase activity, DSB resection is mechanistically more alike that in eukaryotes (by its uncoupling from a recombinase polymerization step), and remarkably, the role of RecQ also becomes akin of its eukaryotic counterparts in a way of promoting homologous and suppressing illegitimate recombination. The sickly phenotype of *recB1080 recQ* mutant was further exacerbated by inactivation of an exonuclease I, which degrades the unwound 3′ tail. The respective *recB1080 recQ xonA* mutant showed poor viability, DNA repair and homologous recombination deficiency, and very increased illegitimate recombination. These findings demonstrate that the metabolism of the 3′ ending overhang is a decisive factor in tuning the balance of homologous and illegitimate recombination in *E. coli*, thus highlighting the importance of regulating DSB resection for preserving genome integrity. *recB* mutants used in this study, showing pronounced RecQ helicase and exonuclease I dependence, make up a suitable model system for studying mechanisms of DSB resection in bacteria. Also, these mutants might be useful for investigating functions of the conserved RecQ helicase family members, and congruently serve as a simpler, more defined model system for human oncogenesis.

## Introduction

DNA is an inherently stable and inert molecule when in duplex form. However, for its biological functions to be fulfilled it needs to be temporarily and site-specifically activated. Activation is achieved by means of creating a single-stranded (ss) intermediate, which enables DNA metabolic processes such as transcription, replication, homologous recombination and repair etc.

During homologous recombination ssDNA is a substrate onto which a recombinase protein (RecA, RadA, Rad51 (Dmc1) from bacteria, archaea and eukaryotes, respectively) is loaded, which then catalyzes an invasion of a homologous duplex DNA, giving rise to a joint molecule structure, named D-loop [1]. In that way DNA gets modified through exchanges with homologous sequences, but also preserved as this process repairs DNA lesions such as double strand breaks (DSBs) and impaired replication forks [1].

Cellular helicases and nucleases produce 3′-ending ss tails when processing DSBs, a process known as a DNA end resection, while internal ss regions, gaps, usually originate from replication defects [1]. When ssDNA appears in a cell, it is bound by a specific protein designated SSB in bacteria and RPA in eukaryotes. These proteins orchestrate action of many other proteins on ssDNA, while preventing recombinase polymerization [2]. This recombination inhibition is overcome by the action of dedicated proteins called recombination mediator proteins (RMPs), which catalyze an exchange of ss-binding protein for its cognate recombinase protein [3]. The main RMPs in bacteria are RecFOR and RecBCD/AddAB proteins [4]. While RecFOR proteins catalyze an SSB-RecA exchange on gapped DNA at the ssDNA-dsDNA junction [5], RecOR complex binds to an SSB-ssDNA structure and mediates an exchange of SSB for RecA [6]. RecBCD/AddAB class of enzymes binds with high affinity to a flush dsDNA end and unwinds it with its fast and processive helicase activity (reviewed in [7]). The enzyme degrades the unwound strands, until it encounters a specific octanucleotide sequence named χ. Interaction with χ changes RecBCD, which ceases degradation of the 3′-ending strand while increasing degradation of its 5′-ending complement. Concomitantly, a χ-modified RecBCD facilitates RecA loading onto thus created 3′-ending tail [8]–[10].

There are some situations in *E. coli* in which RecFOR proteins catalyze RecA polymerization onto ssDNA originating from a dsDNA end. This happens in a *recBC* null mutant that is also deficient in exonucleases exonuclease I (ExoI) and SbcCD, which degrade 3′-ending tails. Here, RecQ helicase produces ss tails and RecFOR proteins enable RecA loading onto the 3′-ending one, while its complementary strand is degraded by a RecJ exonuclease [1]. Another example is a “hybrid” recombination pathway [11], which occurs in mutants devoid of RecBCD’s nuclease and RecA loading activities due to a single amino acid change in an active center of the unique nuclease and RecA loading domain of the RecB subunit (*recB1080* and *recB1067* mutations) [12]–[15]. Here, intact DNA binding and helicase activities of RecB^1080^CD (and RecB^1067^CD) enzyme are coupled with an exonuclease activity of RecJ exonuclease (degrading the 5′-ended unwound strand) and a RecA loading activity of RecFOR proteins (onto the unwound 3′-ending tail) to promote homologous recombination, as well as UV and γ-survival [14], [16].

Because of its essential role in virtually every organism, homologous recombination is a well conserved and regulated process. When regulation of homologous recombination is compromised, an aberrant, illegitimate recombination ensues, occurring between regions of limited, or of no homology [17]. Illegitimate recombination leads to a genome instability, which causes a poor viability in viruses, bacteria and eukaryotes, and also cancer, sterility and many other illnesses in vertebrates.

DNA helicases of the RecQ family are critical for preserving genome integrity, hence their classification as “caretakers” of the genome [18]. RecQ helicases are highly conserved in evolution, both functionally and structurally, from bacteria to humans [19]. In unicellular organisms one family member is found in each organism (e.g. RecQ in bacteria and Sgs1 in budding yeast, Rqh1 in fission yeast), whereas there are as many as five human analogs: RECQL, RECQ4, RECQ5, WRN and BLM [19].

Biochemically, they show 3′–5′ polarity of DNA unwinding, with respect to the strand that binds the enzyme [20]. RecQ binds to a variety of substrates including: three and four way junctions (Holliday junctions), both the 3′ and 5′ ssDNA overhangs, a duplex DNA both internally and at the end, and, notably, it binds to recombination intermediates, joint molecules in Kappa and D-loop formation [21]. Such a broad substrate specificity allows RecQ helicase to act both presynaptically to promote homologous recombination in bacteria (as described above) as well as in humans [22] and yeast [23], and postsynaptically to decatenate junctions and disrupt nascent joint molecules, especially those arising from aberrant, nonhomologous joint exchanges [21], [24], [25].

A physiological effect of RecQ activity is seen in humans where deficiency in RecQ helicases WRN, BLM and RECQ4 give rise to recessive disorders Werner syndrome, Bloom syndrome and Rothmund-Thompson syndrome (as well as RAPADILINO and Baller-Geller syndromes), respectively [18]. All these disorders are characterized by a cancer predisposition and an increased genomic instability, the latter also being a phenotype in yeast Sgs1 mutants, along with accelerated ageing [18].

However, considering the adversity of effects rendered by inactivating a single out of five RecQ analogs in humans, it is quite remarkable that a complete RecQ deficiency in wild-type (wt) *E. coli* has barely detectable effects. Although it causes an increase in illegitimate recombination [26], it has no effect on cell viability, DNA repair and homologous recombination [27], [28].

The aim of this study was to characterize the factors responsible for such different phenotypes of RecQ deficient *E. coli* and humans. The recombinogenic substrates, dsDNA ends, are processed in wt *E. coli* exclusively by RecBCD enzyme which, for having no eukaryotic analogue, we considered the most likely differentiating factor in a recombination regulation between *E. coli* and humans. Since RecBCD is unique among DSB resection machines for possessing a recombinase loading activity, we made use of the aforementioned mutant RecB^1080^CD and RecB^1067^CD enzymes, exhibiting only a helicase activity, to uncouple creation of a long, reactive 3′-ending overhang from RecA polymerization onto it, and hence to make a DSB resection in *E. coli* more similar to that in eukaryotes. Inactivation of RecQ helicase in *recB1080* and *recB1067* mutants indeed caused a sickly phenotype consisting of a decreased cell viability and a reduced DNA repair and homologous recombination efficiency. At the same time, an illegitimate recombination frequency strongly increased in a *recB1080 recQ* mutant. The effects of the *recQ* mutation were exacerbated by a *xonA* mutation, inactivating a processive exonuclease, ExoI, that degrades 3′-ending tails [29], suggesting that the long 3′-ending tails produced by the mutant RecBCD are shifted from homologous recombination reactions into the nonproductive, aberrant DNA exchanges. We conclude that in *E. coli* the recombinogenic 3′ tails are channeled into productive reactions and away from aberrant ones by the coupled helicase and RecA loading activities of RecBCD enzyme and, upon their uncoupling, by the concerted action of RecQ helicase and ExoI.

## Results

### RecQ Helicase and ExoI are Required for DNA Repair in *recB* Mutants Deficient in Nuclease and RecA Loading Activities

**Figure 1:**
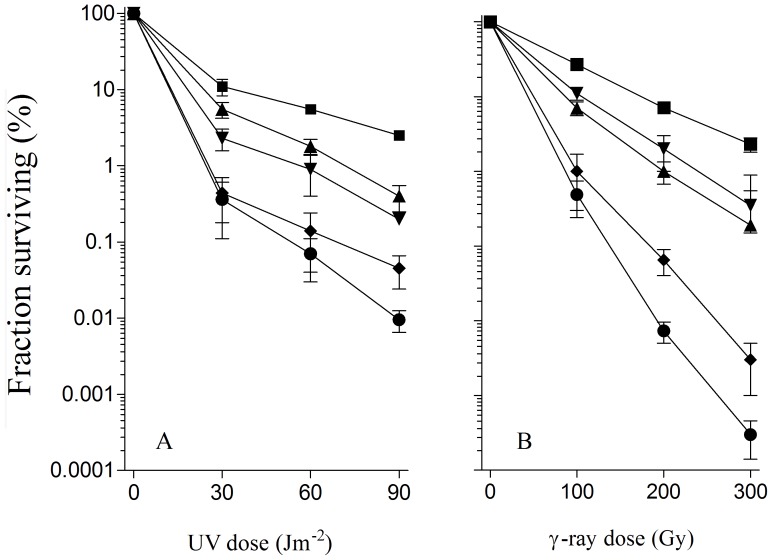
*ΔxonA* and *ΔrecQ* mutations impair DNA repair in UV irradiated (A) and γ-irradiated (B) *recB1080* mutant cells, which lack nuclease and RecA loading activities of RecBCD. Fraction survival is given as a fraction of the unirradiated control. Symbols: (▪) *recB1080*; (▴) *ΔxonA recB1080*; (▾) *ΔrecQ recB1080*; (♦) *ΔxonA ΔrecQ recB1080*; (•) *recB268*::Tn*10*.

**Figure 2:**
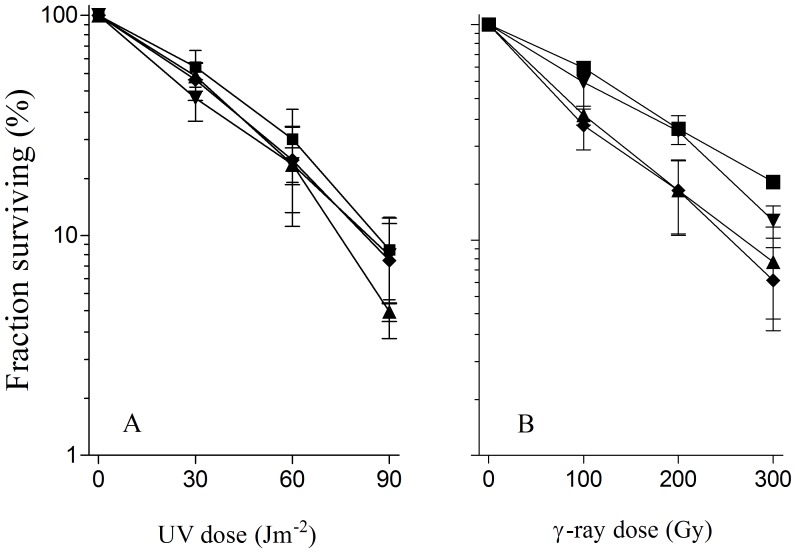
DNA repair in UV irradiated (A) and in γ-irradiated (B) wild-type bacteria is mostly unaffected by *ΔxonA* and *ΔrecQ* mutations. Fraction survival is given as a fraction of the unirradiated control. Symbols: (▪) AB1157 *rec^+^*; (▴) *ΔxonA*; (▾) *ΔrecQ*; (♦) *ΔrecQ ΔxonA*.

By using P1 transduction we introduced a *recQ* deletion into *recB1080* and *recB1067* mutants, as well as in wt strain, and assessed their DNA repair capacity. DNA repair was evaluated by measuring UV and γ-survival of the mutants. As shown in Figure 1, the *recB1080 ΔrecQ* mutant was considerably more sensitive to both UV and γ-irradiation than the parental *recB1080* strain. At the highest UV and γ-dose tested, the survival of the double mutant was about 10 fold lower than that of the parent strain. A *Δ recQ* derivative of the *recB1080* mutant was about two fold more radiosensitive than *recB1080*, when comparing survival slopes (Figure 1B). The same effect was observed in *recB1067* background (not shown). Although our results are somewhat similar to those of a previous study, the conclusion of that study (that RecQ is “not needed for RecB1080CD-mediated recombination”) [16] is opposite to our conclusions. As expected, and in accord with previous studies [27], [28], RecQ inactivation did not produce any effect on UV and γ-survival of otherwise wt cells (Figure 2).

In order to determine the role of the unwound 3′ tail processing in *recB1080*, we inactivated ExoI, the most powerful exonuclease that acts on 3′ overhangs in *E. coli* [29]. Much like a *recQ* deletion, an ExoI inactivation rendered a resultant *recB1080 ΔxonA* mutant substantially more sensitive to UV and γ-irradiation compared to the parental *recB1080* mutant (Figure 1). Essentially the same results were obtained with a *ΔxonA* derivative of *recB1067* mutant. It was also notably more sensitive to UV and γ-irradiation than its parental strain (data not shown). On the other hand, a *ΔxonA* derivative of a wt strain (AB1157) was as UV resistant as its parental strain, while being slightly more radiosensitive than AB1157 (Figure 2), indicating that ExoI function is required much more for DNA repair in the nuclease and RecA loading deficient *recB* mutants than in wt bacteria.

Next, we inactivated genes coding for RecQ and ExoI in *recB1080* and wt strains and noticed opposite effects. Whereas in the wt background no effect was seen on both UV and γ-survival (Figure 2), a *recB1080 ΔrecQ ΔxonA* mutant was extremely sensitive to both agents, almost as a *recB* null mutant DE101 (Figure 1). As expected, a *ΔxonA ΔrecQ recB1067* mutant was also almost completely devoid of DNA repair (data not shown), suggesting that DNA repair in mutants deficient in RecBCD’s nuclease and RecA loading activities depends heavily on the functions of ExoI and RecQ proteins. The synergism of ExoI and RecQ suggests that they act in parallel, overlapping pathways in that background.

Conversely, a pSQ211 plasmid, carrying a *recQ* ^+^ gene, rendered the *ΔrecQ recB1080* mutant as resistant to UV irradiation as the single *recB1080* mutant, while having no effect on UV survival of the *ΔxonA recB1080* mutant (data not shown). When introduced into the triple *ΔxonA ΔrecQ recB1080* mutant, the plasmid complemented its UV sensitivity only partially, to the level of double mutants (data not shown). These results suggest that the RecQ helicase supplied *in trans* complements only a missing RecQ helicase but not an ExoI activity in the *recB1080* mutant background.

### RecQ Helicase and ExoI are not Required for DNA Repair in Nuclease Deficient but RecA Loading Proficient *recB* Mutants

**Figure 3:**
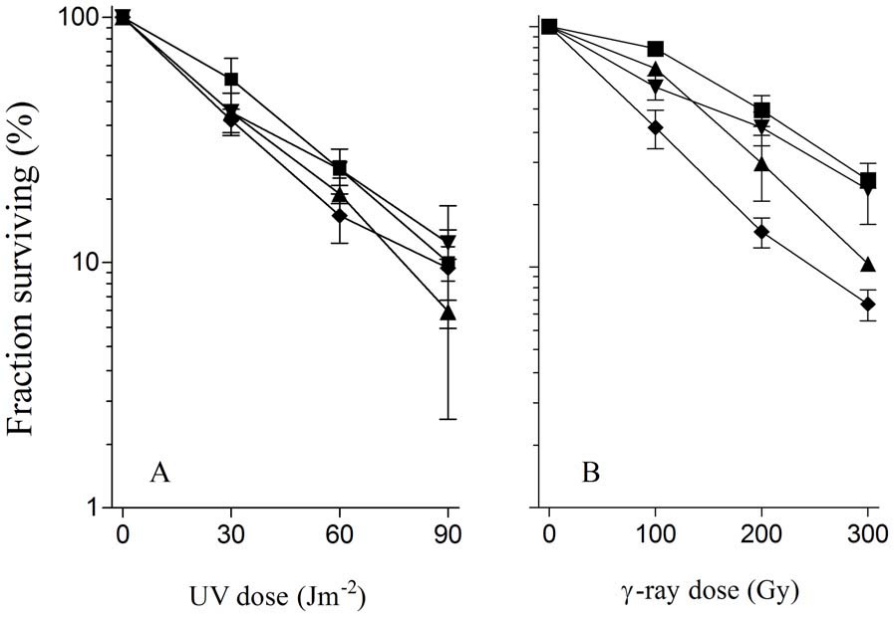
Inactivation of *xonA* and *recQ* genes does not affect UV radiation survival (A) and γ-survival (B) of nuclease deficient but RecA loading proficient *recB1080 recD* mutants. Fraction survival is given as a fraction of the unirradiated control. Symbols: (▪) *recB1080 recD*; (▴) *recB1080 recD ΔxonA*; (▾) *recB1080 recD ΔrecQ*; (♦) *recB1080 recD ΔrecQ ΔxonA*.

When the RecB^1080^CD and RecB^1067^CD mutant enzymes, devoid of both nuclease and RecA loading activities [14], [30], lack the RecD subunit (in *recD* derivatives of *recB1080* and *recB1067*), they gain the ability to constitutively load RecA protein, while remaining nuclease deficient [14], [30]. We used the *recD recB1080* and *recD recB1067* mutants to determine which of the two missing RecBCD functions is/are bypassed with the functions of ExoI and RecQ helicase. When either single *ΔxonA* or *ΔrecQ* mutations, or both of them, were introduced into a *recD* derivative of the *recB1080* mutant, they did not affect its UV and γ-survival (Figure 3). The same behavior was observed in the *recD recB1067* mutant; introduction of either the *ΔxonA* or the *ΔrecQ* or both of these mutations did not influence its UV and γ-survival (data not shown). These results corroborate earlier findings that *recQ* [31] or *xonA* [32] mutation does not affect UV and γ-survival in *recD* derivatives of *recB* nuclease deficient strains. An individual *ΔxonA* or *ΔrecQ* mutation, as well as their combination, also did not change UV and γ-survival of RIK144, a *recD* derivative of the wt strain (not shown). These results show that ExoI and RecQ helicase are not required for DNA repair in the nuclease deficient but RecA loading proficient *recD recB* mutants; meaning that RecB^1080^C-catalyzed RecA loading at DNA ends renders the two enzymes dispensable.

### RecQ Helicase and ExoI are Required for Maintaining Cellular Viability in Nuclease and RecA Loading Deficient *recB* Mutants

Exogenous genotoxic agents (*e.g.* UV and γ) are thought to pose only a minor, occasional threat to a cell, wherein the majority of DNA damage stems from a continuous action of endogenous genotoxic agents such as reactive oxidative species and various replication impediments [1]. The ability of *E. coli* to cope with an endogenously caused DNA damage is assessed genetically by determining its viability. A cell is considered viable when able to give rise to a colony.

A colony-forming ability of the nuclease and RecA loading deficient *recB1080* mutant was about one third lower than that of the wt strain, and was further decreased by one third and half upon introducing *ΔrecQ* and *ΔxonA* mutations, respectively (Table 1). *ΔxonA* and *ΔrecQ* mutations exhibited a synergistic effect rendering the *ΔxonA ΔrecQ recB1080* mutant poorly viable. Its viability of about 13% was about 8 and 5 fold lower than that of the wt and the *recB1080* mutant, respectively (Table 1), meaning that, on average, only one out of eight *ΔxonA ΔrecQ recB1080* cells was able to give rise to a colony. As a consequence, this mutant grew slowly in a liquid medium and on agar plates. As expected, a colony-forming ability of the *ΔxonA ΔrecQ recB1067* mutant was similar to that of the *recB1080 ΔxonA ΔrecQ* (data not shown). The viability of the triple *ΔxonA ΔrecQ recB1080* mutant increased to about 32% when harboring the RecQ-producing plasmid.

**Table 1:** Relative cellular viability and conjugational recombination proficiency of *ΔrecQ* and *ΔxonA* derivatives of wild-type, *recB1080*, *recD* and *recD recB1080* strains.

Strain	Relevant genotype	Relative viability^a^	Recombination frequency^b^
AB1157	*rec^+^*	1.0	1.0
DE110	*ΔrecQ*	0.99±0.014	1.0±0.23
DE120	*ΔxonA*	0.98±0.022	0.47±0.032
DE130	*ΔrecQ ΔxonA*	0.98±0.047	0.44±0.055
RIK174	*recB1080*	0.64±0.076	0.18±0.045
DE150	*recB1080 ΔrecQ*	0.43±0.096	0.097±0.024
DE151	*recB1080 ΔxonA*	0.30±0.039	0.023±0.008
DE152	*recB1080 ΔrecQ ΔxonA*	0.13±0.025	0.014±0.003
RIK144	*recD*	1.03±0.011	2.18±0.52
DE160	*recD ΔrecQ*	0.95±0.07	1.62±0.14
DE161	*recD ΔxonA*	0.84±0.07	0.33±0.126
DE162	*recD ΔrecQ ΔxonA*	0.84±0.028	0.23±0.03
DE169	*recB1080 recD*	0.88±0.072	2.00±0.24
DE170	*recB1080 recD ΔrecQ*	0.92±0.07	1.43±0.34
DE171	*recB1080 recD ΔxonA*	0.90±0.013	0.25±0.087
DE172	*recB1080 recD ΔrecQ ΔxonA*	0.81±0.093	0.36±0.17
DE101	*recB268*	0.33±0.064	0.012±0.006

a Wild-type cellular viability of 1.0 corresponds to 1.3×10^8^ of colony formers per ml at OD_600_ of 0.3.

b Wild type frequency of 1.0 corresponds to 11.3 Pro^+^ transconjugants per 100 Hfr3000 cells. Recombination frequency was corrected for the viability of recipients.

The observed low colony-forming ability of the triple mutant might be due either to its low cellular viability or a filamentation. In order to differentiate between these two possibilities, we examined *ΔxonA ΔrecQ recB1080* mutant cells microscopically and observed only a few filaments in their population (not shown). We conclude, therefore, that the low colony-forming ability of the triple mutant reflects its low cellular viability.

On the other hand, cellular viability of wt, *recD* and *recD recB1080* mutants (as well as that of *recD recB1067*, not shown), was not affected by *ΔxonA* and *ΔrecQ* mutations (Table 1). These results are in accord with previous reports showing that *recQ* inactivation does not render wt and *recB1080 recD* cells less viable [27], [31], and also a *xonA* mutation does not affect a viability of *recD* and *recB1067 recD* mutants [32].

Overall, these results suggest that gene products of *xonA* and *recQ* are required for maintaining cellular viability of the *recB* mutants deficient in nuclease and RecA loading activities, but not in their *recD* derivatives (deficient only in nuclease activity) nor in wt strain and its *recD* derivative**.**

### RecQ Helicase and ExoI Promote Homologous Recombination in Nuclease and RecA Loading Deficient *recB* Mutants

Another important DNA metabolic process in *E. coli* that depends on RecBCD enzyme is homologous recombination. We used Hfr conjugational and P1 transductional crosses to determine the recombination proficiency of the mutants mentioned above. Table 1 shows that, in accord with the DNA repair data, either a *xonA* or a *recQ* inactivation impairs conjugational homologous recombination in the *recB1080* mutant, with the *xonA* mutation exerting stronger effect. The triple *ΔxonA ΔrecQ recB1080* mutant was even more recombination deficient; it recombined DNA practically with the same efficiency as the *recB* null mutant (Table 1). Similar effects of *ΔxonA* and *ΔrecQ* mutations on homologous recombination as those described above were observed in the *recB1067* background (data not shown), in contrast to earlier reports where no effect of a *xonA* mutation on recombination of λ phage in *recB1067* [14] and a *recQ* mutation on conjugational recombination in *recB1080* mutant was observed [16].

Again, similarly to the DNA repair data mentioned above and in accord with earlier studies [27], [31], the *recQ* mutation did not affect conjugational recombination proficiency of wt, *recD* and *recD recB1080* strains (as well as that of *recD recB1067*, data not shown) (Table 1). The *xonA* mutation reduced moderately the efficiency of homologous recombination in *recD* and *recD recB1080* mutants (and also in *recD recB1067*, data not shown), in accord with an earlier report [32], whereas recombination in wt bacteria was even less affected (Table 1), corroborating an earlier study [33]. A moderate effect of the *xonA* mutation in wt, *recD* and *recB1080 recD* genetic backgrounds was not increased by the *recQ* mutation (as in *recB1080* strain) (Table 1), suggesting that ExoI and RecQ do not act synergistically in these strains.

Since 3′–5′ ssExos, including ExoI, show a specific role in transferred DNA processing during an Hfr-mediated conjugation [32], we used P1 transduction crosses in order to determine the role of ExoI and RecQ in homologous recombination reactions in a simpler and more straightforward way. The frequency of Pro^+^ transductants was 0.313 in the *recB1080* mutant, compared to AB1157 strain. *xonA*, *recQ*, and *xonA recQ* derivatives of *recB1080* exhibited recombination frequency 0.106±0.0075, 0.127±0.007 and 0.037±0.005, respectively, relative to the wt strain. *xonA recQ* and *recB1080 recD xonA recQ* mutants produced 0.635±0.07 and 0.91±0.044 transductants compared to their respective parental strains AB1157 and DE169 (*recB1080 recD*).

We may thus conclude that the requirement for RecQ and ExoI functions in homologous recombination in *E. coli* is much more pronounced in mutants exhibiting only a helicase activity of the RecBCD than in those showing all three activities of the enzyme (wt cells), or those showing helicase and RecA loading activities (*recD* mutants), which is in complete accord with their role in a DNA repair and viability maintenance.

### Synergistic Action of RecQ Helicase and ExoI Suppresses Illegitimate Recombination in Nuclease and RecA Loading Deficient *recB1080* Mutant

During λ phage infection, a viral genome gets integrated into an *E. coli* chromosome between *gal* and *bio* genes, thus establishing a lysogenic mode of infection. Upon λ prophage induction, its genome is excised from a bacterial chromosome in a generally precise reaction. However, the excision is sometimes aberrant, caused by an illegitimate recombination event, and hence a neighboring part of the bacterial genome is packed into λ’s head instead of part of its own genome [34]. When during a λ*cI857* prophage thermoinduction excised phage carries a part of the *bio* gene containing a χ sequence, part of its genome coding for *red* and *gam* functions is deleted. Such a λ*bio* phage, excised by illegitimate recombination, gives rise to a Spi^−^ phenotype [35]. That means it is capable of growing on a P2 lysogenic *E. coli*, whereas wt λ is not. Therefore, the frequency of Spi^−^ phage arising from λ*cI857* prophage induction represents a frequency of illegitimate recombination [35].

We measured the appearance of Spi^−^ phage in wt and *recB1080* backgrounds. Very low frequency of Spi^−^ phage originating from wt cells was increased about 13 fold in *recQ* mutant (Table 2), which is close to the lower limit of the range observed earlier [26]. Inactivation of ExoI increased appearance of Spi^−^ phage only slightly (Table 2). In our assay, therefore, we did not observe an effect of ExoI on illegitimate recombination in wt bacteria, in contrast to earlier studies (using different assays) which found that ExoI suppresses illegitimate recombination in that background [36], [37]. A double *recQ xonA* mutant produced Spi^−^ phage at a rate similar to a *recQ* single mutant, i.e. it was increased about 18 fold (Table 2).

**Table 2:** Illegitimate recombination is suppressed in *recB1080* mutant by synergistic activities of RecQ helicase and ExoI exonuclease.

Strain	Relevant genotype	Burst size	λ Spi^−^ frequency^a^ (×10^−10^)^b^	Relative rate^c^
DE105	rec^+^ λ*cI*857	35	3.62±1.60(55.0)	1.0(15.2)
DE111	*ΔrecQ* λ*cI*857	32	45.66±18.87(330.0)	12.6(7.2)
DE121	*ΔxonA* λ*cI*857	31	5.66±4.04(277.5)	1.56(48.9)
DE131	*ΔxonA ΔrecQ* λ*cI*857	41	64.00±46.13(1225.0)	17.7(19.1)
DE153	*recB1080* λ*cI*857	64	16.67±5.77(1975.0)	4.6(118.5)
DE154	*recB1080 ΔrecQ* λ*cI*857	64	320.00±105.83(2750.0)	88.4(8.6)
DE155	*recB1080 ΔxonA* λ*cI*857	78	336.67±271.35(3150.0)	93.0(9.4)
DE156	*recB1080 ΔrecQΔxonA* λ*cI*857	87	1410. 00±85.44(3950.0)	389.5(2.8)
DE173	*recB1080 recD* λ*cI*857	57	10.00±0.09	2.76
DE174	*recB1080 recD ΔrecQ* λ*cI*857	43	440.00±124.90	121.5
DE175	*recB1080 recD ΔxonA* λ*cI*857	73	8.33±2.89	2.3
DE176	*recB1080 recD ΔrecQ ΔxonA* λ*cI*857	48	310.00±252.39	85.6
DE102	*recB268* λ*cI*857	32	6.66±2.89(1375.0)	1.83(206.4)

a λ Spi^−^ frequency was calculated by dividing the titer of λ Spi^−^ phage by the titer of total phage.

b λ Spi^−^ frequency in bacteria irradiated with 30 Jm^−2^ dose of UV light. The values are averages of two independent experiments, and are shown in brackets.

c Illegitimate recombination frequency was expressed relative to the wild-type strain AB1157. For each UV-irradiated bacterial culture, recombination is expressed in relation to its unirradiated part, and is shown in brackets.

The nuclease and RecA loading deficient *recB1080* mutant yielded somewhat higher number of Spi^−^ phage, frequency of which was increased about five fold compared to wt strain (Table 2). This suggests that processive helicase activity of the mutant RecB^1080^CD enzyme, devoid of nuclease and RecA loading activities, leads to an increased illegitimate recombination. Inactivation of RecQ helicase in *recB1080* mutant increased the Spi^−^ phage frequency about 20 and 90 fold, compared to its parental and wt strain, respectively (Table 2). About the same effect was observed in a *xonA* derivative of the *recB1080* mutant (Table 2). Furthermore, when both *recQ* and *xonA* mutations were introduced into *recB1080* strain, their synergistic effect was observed and Spi^−^ phages were produced about 85 fold more often than in the parental strain, and about 390 fold more frequent than in wt strain.

We also determined a Spi^−^ phage yield in a *recD* derivative of *recB1080*, wherein RecB^1080^C enzyme exhibits coupled helicase and RecA loading activities. The slightly increased rate of a Spi^−^ phage occurrence in *recB1080 recD* strain was further increased about 40 fold by the *recQ* mutation (Table 2). On the other hand, the *xonA* mutation did not exert any effect on a Spi^−^ phage production in the *recB1080 recD* genetic background. The *recB1080 recD recQ xonA* mutant produced somewhat less Spi^−^ phage than the *recB1080 recD recQ* strain (Table 2). The *recB1080 recD* strain is therefore similar to the wt strain by the way of RecQ suppressing illegitimate recombination and ExoI not having any effect on it. In contrast, illegitimate recombination in the *recB1080* mutant is suppressed by synergistic action of RecQ and ExoI. However, the fact that illegitimate recombination is still increased in that mutant despite intact activities of RecQ and ExoI, suggests that their combined suppressing capacity is exceeded.

In an earlier study [35] an illegitimate recombination frequency was increased in UV irradiated *E. coli*. We irradiated wt and *recB1080* strains as well as their *recQ, xonA* and *recQ xonA* derivatives in order to determine the overall cellular capacity for illegitimate recombination. The UV irradiated cells indeed gave rise to more Spi^−^ phage than their unirradiated counterparts. The increase in a Spi^−^ phage production ranged from 7.2 to 48.9 fold in the wt background and from 2.8 to 118.5 fold in the *recB1080* background (Table 2). Surprisingly, the *recB1080 recQ xonA* mutant, suffering the highest spontaneous illegitimate recombination, had the lowest Spi^−^ phage frequency increase upon UV irradiation (just 2.8 fold) (Table 2), suggesting that a limit of illegitimate recombination occurrence in *E. coli*, or in our assay sensitivity, is met. Strains exhibiting a lower rate of spontaneous illegitimate recombination often had the higher increase upon UV irradiation, coming closer to a maximal illegitimate recombination frequency of about 4×10^−7^ (Table 2). This saturation point is about 1100 fold above the level in unirradiated wt strain.

Overall, our results suggest that when a highly processive and fast helicase activity of RecBCD enzyme is uncoupled from its RecA loading activity, a long 3′ tail hence produced is very prone to engaging in aberrant, nonproductive exchanges, thus leading to illegitimate recombination and away from homologous recombination events. This activity is counteracted by a 3′–5′ helicase activity of RecQ and a 3′–5′ exonuclease activity of ExoI, which thereby enable homologous and suppress illegitimate recombination in the *recB1080* background.

## Discussion

RecBCD, a multifunctional bacterial enzyme, belongs to the common DNA-end resecting machinery consisting of a helicase-nuclease tandem. A combination of a helicase and a 5′–3′ exonuclease resects DSBs in: bacteria (RecBCD, RecQ/RecJ), archaea (HerA/NurA), yeast (Sgs1/Dna2), *Xenopus laevis* (xWRN/xDNA2), humans (BLM/EXOI), etc. [38]. However, RecBCD is unique for having a recombinase loading activity, which, together with the enzyme’s helicase and nuclease activities, produces a RecA nucleofilament in a highly coordinated manner.

In order to make a DSB resection in *E. coli* more similar to that in eukaryotes and archaea, we made use of the *recB1080* and *recB1067* mutants, coding for the RecB^1080^CD and RecB^1067^CD enzymes, respectively, that possess a fast and processive helicase activity, but are devoid of nuclease and RecA loading activities (as no difference was observed between the two mutants, *recB1080* will be discussed hereafter, referring also to *recB1067*). A helicase/exonuclease pair RecB^1080^CD/RecJ, operative in that mutant [14], is therefore functionally analogous to budding yeast Sgs1/Dna2, *Xenopus laevis* xWRN/xDNA2 and human BLM/EXOI helicase/exonuclease pairs that catalyze a long-range DSB resection step during homologous recombination repair in those organisms [38].

The RecB^1080^CD/RecJ pair is not as efficient as RecBCD since *recB1080* mutant exhibits reduced cell viability, an impaired DNA repair and homologous recombination, but an increased illegitimate recombination. We show here that RecQ helicase is required for maintaining cell viability, DNA repair and homologous recombination in the *recB1080* mutant, but also for suppressing illegitimate recombination. These results indicate a postsynaptic, disruptase role for RecQ in controlling the fidelity of recombination exchanges, i.e. funneling the recombinogenic 3′ tails produced by RecB^1080^CD into productive DNA exchanges and away from the aberrant ones. This kind of behavior mimics that observed in eukaryotes, where RecQ analogues were shown to both promote homologous recombination and suppress illegitimate recombination and genomic instability that may lead to cancer [18].

We also inactivated ExoI, the principal *E. coli* 3′–5′ exonuclease [29], in order to increase longevity of the recombinogenic 3′ tails, and show that the resultant *recB1080 xonA* strain displays similar pleiotropic effects as its *recQ* counterpart. ExoI and RecQ act in synergy since the triple *recB1080 recQ xonA* mutant is poorly viable and is almost completely devoid of homologous recombination and DNA repair, yet with ∼400 fold increased illegitimate recombination, compared to the wt strain. In that strain 3′ overhangs are directed into aberrant, nonproductive exchanges (increased ∼400 fold); instead of in productive reactions (decreased ∼30–100 fold).

Since the shift from homologous to illegitimate recombination was not observed in *recQ* and *xonA* derivatives of wt and *recB1080 recD* cells, exhibiting helicase/nuclease/RecA loading and helicase/RecA loading activities respectively, we conclude that this shift in the *recB1080* mutant is due to a fast and processive helicase activity of RecB^1080^CD, lacking a coordinated RecA polymerization activity. Hence, the differences in phenotypes of *E. coli* and humans deficient in RecQ and its eukaryotic analogs, respectively, are not due to differences among these enzymes but rather to differences in recombination substrates metabolisms in these organisms. A robust RecBCD action at DSBs, which fixes them efficiently through homologous recombination, does not leave much space for the RecQ function in wt *E. coli*. Accordingly, when RecBCD function is affected by an overexpression of RecET proteins in otherwise wt cells, DSB processing becomes increasingly aberrant, leading to increased illegitimate recombination. In these cells RecE nuclease, along with RecT, RecJ and RecFOR proteins, promotes illegitimate recombination while RecQ suppresses it (reviewed in [17]).

The synergism of a 3′–5′ helicase activity of RecQ and a 3′–5′ exonuclease activity of ExoI in the *recB1080* mutant, which we revealed in this study, mimics the activities of human and *X. laevis* WRN and xWRN RecQ analogues, respectively, which are both 3′–5′ helicases and 3′–5′ exonucleases [18]. They act in both homologous recombination initiation and in preventing aberrant exchanges [39], [40], and when WRN protein lacks helicase and exonuclease activities, the cancer-prone Werner syndrome ensues [41], which is characterized by an increased genomic instability and reduced homologous recombination [41].

The evolutionary conservation of a RecQ family function is exemplified by the ability of human BLM protein to partially suppress illegitimate recombination in a *recQ* mutant of *E. coli*, as assessed by the λ Spi^−^ assay [42], which we used in this study too. This suppression indicates that substrate specificity is conserved from RecQ to BLM, and that illegitimate recombination events detected by the λ Spi^−^ assay are similar enough to those occurring in human cells to fall into range of BLM substrate specificity. This should not be surprising since illegitimate recombination events observed in that assay stem from resected DSBs (3′ overhangs) caused by replication impairment [17], which is a common challenge to genome integrity in living organisms [1].

In fact, when comparing an illegitimate recombination pathway (IR) observed in the λ Spi^−^ assay in *E. coli* with eukaryotic DSB repair pathways, one can notice that the IR bears resemblance with microhomology-mediated end joining (MMEJ). Both pathways are initiated by DSB resection, giving rise to 3′ overhangs, which align broken ends by an end-joining reaction dependent on microhomologies (∼4 nt for MMEJ and ∼9 nt for IR) and a ligase function [17], [43]. Both pathways are recombinase independent and are suppressed by homology dependent repair, which is regulated at a DSB resection step [43], [17]. Although known for some time, MMEJ has only recently been appreciated as the “major mechanism for chromosome translocations, and possible other rearrangements, in mammalian cells” [43]. Recurrent chromosome translocations are found in many hematological, epithelial malignancies, also in solid tumors etc. [43]. A key factor in generating these tumorigenic rearrangements appears to be the extent of DNA resection [43].

The conservation of RecQ helicase family functions, DNA damage inducing mechanisms, as well as aberrant recombination intermediates between *E. coli* and humans, allows the *recB1080* mutant and the λ Spi^−^ assay to make up a suitable bacterial model for studying RecQ helicase family functions, especially that of WRN and BLM proteins, in order to elucidate the molecular bases of genomic instability and its malignant phenotypes.

Our results show that DSB resection is a critical factor determining the balance of homologous and illegitimate recombination in *E. coli* and, hence, its genome stability. Remarkably, when *E. coli* DSB resection becomes mechanistically similar to that in eukaryotes (by its uncoupling from a DNA recombinase polymerization step), the role of RecQ increases and also becomes similar to that of its eukaryotic counterparts in a manner of propagating homologous and preventing illegitimate recombination events.

Another analogy between *E. coli* and eukaryotic DSB resection arises from this study, namely the adverse effect of increase in its processivity on DNA repair efficiency and genome stability. Just as RecBCD, the RecB^1080^CD enzyme is a fast (∼1–2 kbp s^−1^) and very processive helicase [7] (≥50 kbp/binding event, our estimation from the fact that wt RecBCD efficiently unwinds a 50 kbp long χ-less F plasmid fragment and a part of chromosomal DNA before encountering a χ site during Hfr-conjugation, [44]). Since *in vitro* the enzyme is not inactivated upon interaction with χ (as is its wt counterpart), but just slowed down [13], its processivity is certainly higher than that of the wt enzyme. Furthermore, as RecB^1080^CD lacks any nuclease activity, it produces full length overhangs (their length being in range of tens of kb), whereas RecBCD produces just a post-χ 3′ tail whose length is likely several kb at most. Lower viability, decreased homologous recombination efficiency and increased sensitivity to genotoxic agents by the *recB1080* mutant, compared to the wt cells, thus correlates well with an increased processivity of DSB resection in that mutant.

A long (≥50 kb) 3′ tail produced by the RecB^1080^CD in a matter of seconds is long-lived, thus posing a threat for a cell, and a great challenge for ExoI with its ∼275 nt s^−1^ [45] rate of 3′-tail resection. ExoI trimming is required to reduce its length, especially since RecFOR-mediated RecA polymerization, active in that strain [16], is shown to take place at the ss-ds DNA junction, i.e. at the 5′ end of that tail [5]. As RecA needs to be present at the 3′ tip of ssDNA in order to make a productive plectonemic joint molecule [46], a long 3′ overhang makes RecA polymerization from 5′ towards 3′ end a lingering process that inefficiently compete with nonproductive, aberrant DNA exchanges, thus shifting recombination from predominantly homologous to illegitimate.

Similar to *E. coli*, an increased DSB resection processivity in eukaryotes leads to sensitivity to DNA damage and genomic instability [38]. For instance, hyperactive Sae2-S267E and CTIP-T847E nucleases in yeast and humans, respectively, which process dsDNA ends with an increased processivity (leaving longer 3′tails), cause an increased ionizing radiation sensitivity and an increased genome instability, leading to gross chromosomal rearrangements [47], [48]. Also, inactivation of a BRCA1-RAP80 complex, which restricts a DSB resection in humans, confers increased ionizing radiation sensitivity and increased genome instability to HeLa cells [49]. An aberrant DSB resection in all these instances impairs balance of DSB repair pathways in yeast and human cells [47]–[49].

Another consequence of an increased dsDNA end resection by a “runaway” helicase activity needs to be taken into an account. Namely, it is related to an SSB protein pool in a cell. There are about 6000 SSB molecules in an *E. coli* cell [50], which comprise 1500 tetramers that can complex about 50–100 kb of ssDNA (1 tetramer per either 35 or 65 nt, [50]). If RecB^1080^CD unwinds 50 kbp of DNA duplex (which is a quite conservative estimate), then 200 kb of ssDNA is produced in a cell during conjugation (i.e. two unwound strands at two ends of a transferred DNA). That means that during conjugation, at least 100–150 kb of an unwound DNA is free of SSB and thus increasingly reactive, and moreover, more resistant to ExoI and RecJ trimming, which is stimulated by SSB (see below). In that respect, exonuclease activities of ExoI and RecJ serve not just to decrease the length of the unwound overhangs, but also to replenish the exhausted SSB pool in a cell, thus enabling their repeating binding to the SSB-free unwound ssDNA. This argument also provides a rationale for coupling of a vigorous nuclease activity to a fast and processive helicase activity of RecBCD enzyme.

**Figure 4:**
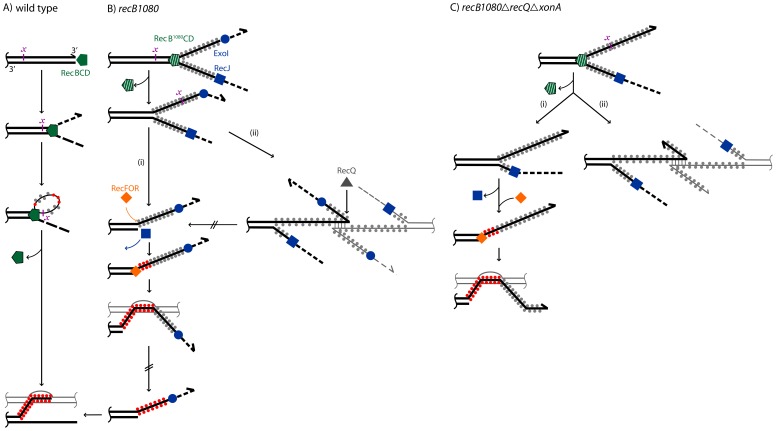
A model for a DSB resection in *E. coli*. Red and gray dots bound on ssDNA tails symbolize RecA and SSB proteins, respectively. Designations (i) and (ii) are assigned to pathways leading to RecA dependent and RecA independent (illegitimate) exchanges, respectively. Hatched arrows represent the nonobligatory, sporadic reaction steps. Details of the reactions are described in the text.

We propose a model for a DSB resection in the *E. coli recB1080* genetic background (Figure 4). As opposed to the situation in wt strain (A), wherein RecBCD enzyme unwinds and degrades both unwound strands upon interaction with a χ site, after which it only degrades the 5′ ending strand while polymerizing RecA onto the 3′ ending strand, thus creating a RecA nucleofilament; in *recB1080* mutant (B), RecB^1080^CD helicase is active. Its fast and processive helicase activity produces long 3′ and 5′ overhangs, onto which SSB molecules are bound. The SSB interacts with ExoI and RecJ ssExos and stimulates their trimming of 3′ and 5′ tails, respectively [51], [52]. Since exonuclease activities of ExoI and RecJ are slower and less processive than the RecB^1080^CD helicase activity, unwound tails are quite stable, which enables them to get involved into nonproductive, aberrant DNA exchanges dependent on a limited homology, thus leading to illegitimate recombination events (ii). However, these aberrant reactions are counteracted by 3′ overhang trimming by ExoI and by a disruptase activity of RecQ helicase, enabling ExoI to further degrade the tail, thus directing it into homologous recombination reactions (i). Homologous recombination is effected by RecFOR-dependent RecA polymerization onto SSB-ssDNA filament, which occurs at an ssDNA-dsDNA junction, i.e. at the 5′ end of the tail. This localization is unfavorable for a RecA filament to catalyze a productive plectonemic joint, and instead an unstable paranemic structure ensues [46]. We propose that this unstable structure is either disrupted by RecQ, as shown for WRN and BLM proteins [18], [40], or by some other factors, thus allowing ExoI and RecA to deliver the RecA nucleofilament to the 3′ tip of the tail and hence direct it into a productive exchange. An alternative RecA polymerization reaction, the one catalyzed by a RecOR complex [6], seems unlikely since *recF*, *recO* and *recR* mutations exert mutually undistinguishable phenotypes in *recB1080* strain [16]. In the *recB1080 recQ xonA* mutant (C), the absence of ExoI function increases the 3′ overhang’s longevity (although ExoVII, SbcCD and ExoX can still act on it); while simultaneously RecQ deficiency increases its reactivity. As a result, nonproductive, aberrant DNA exchanges are stimulated, leading to both RecA independent illegitimate recombination (ii) and RecA dependent paranemic joint molecules (i).

## Materials and Methods

### Strains, Growth Conditions and Media

Designations, genotypes and references/construction of the *E. coli* K-12 strains used in this study are listed in Table 3. Most of them were AB1157 derivatives and were constructed by P1 transduction, as described earlier [53]. Transductants that acquired new allele(s) were selected by new antibiotic resistance(s) they obtained. The genotype *recBD1080A* describes a RecB mutant protein with an amino acid aspartate substituted with an alanine at position 1080 by a point mutation [12], [14]. A plasmid pSQ211 carries the *recQ^+^* gene under a T7 polymerase promoter and overproduces RecQ enzyme upon induction with IPTG [21].

**Table 3:** Bacterial strains used in this study.

Strain	Relevant genotype	Reference or construction
AB1157	Wild type	[55]
DE101	*recB268*::Tn*10*	[54]
DE110	*ΔrecQ*::*kan*	P1.SWM1003 x AB1157 to Kan^r^
DE120	*ΔxonA300*::*cat*	P1.STL2694 x AB1157 to Cm^r^
DE130	*ΔxonA300*::*cat ΔrecQ*::*kan*	P1.SWM1003 x DE120 to Kan^r^
RIK174	*recBD1080A*	[14]
DE150	*recB1080 ΔrecQ*::*kan*	P1.SWM1003 x RIK174 to Kan^r^
DE151	*recB1080 ΔxonA300*::*cat*	P1.STL2694 x RIK174 to Cm^r^
DE152	*recB1080 ΔrecQ*::*kan ΔxonA300*::*cat*	P1.STL2694 x DE150 to Cm^r^
RIK144	*recD1903*::Tn*10d(Tet)*	[14]
DE160	*recD1903*::Tn*10d(Tet) ΔrecQ*::*kan*	P1.SWM1003 x RIK144 to Kan^r^
DE161	*recD1903*::Tn*10d(Tet) ΔxonA300*::*cat*	P1.STL2694 x RIK144 to Cm^r^
DE162	*recD1903*::Tn*10d(Tet) ΔrecQ*::*kan ΔxonA300*::*cat*	P1.STL2694 x DE160 to Cm^r^
DE169	*recB1080 recD1903*::Tn*10d(Tet)*	P1.RIK144 x RIK174 to Tc^r^
DE170	*recB1080 recD1903*::Tn*10d(Tet) ΔrecQ*::*kan*	P1.SWM1003 x DE169 to Kan^r^
DE171	*recB1080 recD1903*::Tn*10d(Tet) ΔxonA300*::*cat*	P1.STL2694 x DE169 to Cm^r^
DE172	*recB1080 recD1903*::Tn*10d(Tet) ΔrecQ*::*kan ΔxonA300*::*cat*	P1.STL2694 x DE170 to Cm^r^
DE105	rec^+^ λ*cI*857	Lysogenization of AB1157
DE111	*ΔrecQ*::*kan* λ*cI*857	Lysogenization of DE110
DE121	*ΔxonA300*::*cat* λ*cI*857	Lysogenization of DE120
DE131	*ΔxonA300*::*cat ΔrecQ*::*kan* λ*cI*857	Lysogenization of DE130
DE153	*recB1080* λ*cI*857	Lysogenization of RIK174
DE154	*recB1080 ΔrecQ*::*kan* λ*cI*857	Lysogenization of DE150
DE155	*recB1080 ΔxonA300*::*cat* λ*cI*857	Lysogenization of DE151
**Strain**	**Relevant genotype**	**Reference or construction**
DE156	*recB1080 ΔrecQ*::*kan ΔxonA300*::*cat* λ*cI*857	Lysogenization of DE152
DE173	*recB1080 recD* λ*cI*857	Lysogenization of DE169
DE174	*recB1080 recD ΔrecQ* λ*cI*857	Lysogenization of DE170
DE175	*recB1080 recD ΔxonA* λ*cI*857	Lysogenization of DE171
DE176	*recB1080 recD ΔrecQ ΔxonA* λ*cI*857	Lysogenization of DE172
DE102	*recB268* λ*cI*857	Lysogenization of DE101
STL2694	*ΔxonA300*::*cat*	[56]
SWM1003	*ΔrecQ*::*kan*	[57]
Hfr3000	Hayes PO1 *proAB^+^*	[55]
NM767	P2 lysogen	Noreen E. Murray

Bacteria were grown in Luria-Bertani (LB) broth and on LB plates [53] at 37°C. Broth and plates used for growing of the mutant strains marked with antibiotic resistance were supplemented with appropriate antibiotics: 20 µg ml^−1^ chloramphenicol, 40 µg ml^−1^ kanamycin, 50 µg ml^−1^ ampicillin and 10 µg ml^−1^ tetracycline. When a strain contained two or three antibiotic markers, antibiotic concentrations were adjusted accordingly.

A fresh overnight culture from a single colony was diluted 100 fold in fresh LB medium supplemented with appropriate antibiotics, when required. Then the culture was grown at 37°C until it reached an optical density at 600 nm (OD_600_) of 0.3. All the experiments were done with log-phase cultures at an OD_600_ of ∼0.3. Unless otherwise stated, each presented value is a mean of at least three independent experiments ± standard deviation.

### UV and γ-irradiations

A previously described procedure was used [54]. Logarithmic cultures were serially diluted in 67 mM phosphate buffer (pH 7.0) and 0.1 ml aliquots spread onto LB plates. The plates were immediately irradiated with 30, 60 and 90 J m^−2^ doses of UV light (254 nm), with a dose rate of 3.0 J m^−2^ s^−1^. A low pressure Hg germicidal lamp was used to irradiate bacteria. The plates were irradiated at room temperature and then incubated in the dark for 24 hours, at 37°C.

For gamma (γ) irradiation, log-phase cultures were exposed to 100, 200 and 300 Gy doses of γ-rays from a ^60^Co source, which provided a dose rate of 10 Gy s^−1^. The γ-irradiations were performed at 0°C, irradiated cells were serially diluted and 0.1 ml samples plated on LB plates. After 24 h of incubation at 37°C, colonies of survivors were scored.

Survival of UV and γ-irradiated bacteria was expressed as a ratio of survivors’ titer and that of total viable, unirradiated cells.

### Cellular Viability

For a cellular viability assay, a logarithmic-phase culture was grown in LB medium at 37°C, with aeration, until reaching an OD_600_ of 0.3. Optical density is a measure of total cell concentration in a population. Then the culture was serially diluted and 0.1 ml aliquots plated on LB plates to determine a number of colony-forming bacteria in a population. Since practically every wt cell is able to develop a colony, a wt culture was a reference for assessing relative viabilities of mutant strains, i.e. what fraction of their total cell titer was able to give rise to colonies.

### Conjugational and Transductional Crosses

Inheritance of the chromosomal Pro^+^ marker was determined in both types of crosses and was expressed relative to wt control strain. The high-frequency recombination (Hfr) conjugational crosses were performed as described by Miller [53]. Log-phase cultures of Hfr3000 donor and recipient cells were mixed at a 1∶10 ratio and allowed to mate for 30 min at 37°C. Then, *proAB* ^+^ transconjugants were selected by plating 0.1 ml samples of a serially diluted mating mixture on minimal M9 plates [53] containing the growth requirements of the recipient except for proline. The plates also contained streptomycin (100 µg ml^−1^) to counterselect donors, and were incubated at 37°C for 24–36 h. Recombination rates in Hfr crosses have been expressed in relation to the titer of donor cells.

Transductions with P1 phage were performed as described earlier [32]. Bacteria were suspended in MC buffer (100 mM MgSO_4_, 5 mM CaCl_2_), infected with P1 at a multiplicity of ∼0.1 and incubated for 30 min at 37°C. Sodium citrate was added to the mixtures, which were then spread onto minimal M9 plates [53] containing 5 mM sodium citrate and the growth requirements of the recipient except for proline.

### Measurement of Frequency of λ Spi^−^ Phage

A procedure developed by Ikeda et al., [35] was used. Strains lysogenic for a thermoinducible λ*cI857* were grown in LB medium at 30°C until reaching an OD_600_ of 0.4. When required, 2 ml of the culture was irradiated with a 30 J m^−2^ dose of UV light (254 nm) from UV crosslinker (Amersham Biosciences), at room temperature. The heat induction of λ prophage was done by incubation at 42°C for 15 min with aeration. The culture was then incubated at 37°C for 2 hours with aeration.

The titer of λ Spi^−^ phage was determined by a P2 lysogenic strain NM767, and the titer of total phage by AB1157. The frequency of λ Spi^−^ phage was obtained by dividing the titer of λ Spi^−^ phage by the titer of total phage. Burst size was calculated by dividing the titer of total phage by the titer of infective centers.
